# Design and Analysis of Multilayered Neural Network-Based Intrusion Detection System in the Internet of Things Network

**DOI:** 10.1155/2022/9423395

**Published:** 2022-09-20

**Authors:** S. K. B Sangeetha, Prasanna Mani, V. Maheshwari, Prabhu Jayagopal, M. Sandeep Kumar, Shaikh Muhammad Allayear

**Affiliations:** ^1^Department of Computer Science and Engineering, SRM Institute of Science and Technology, Chennai, Tamilnadu, India; ^2^School of Information Technology and Engineering, Vellore Institute of Technology, Vellore, TamilNadu, India; ^3^Department of Multimedia and Creative Technology, Daffodil International University, Daffodil Smart City, Khagan, Ashulia, Dhaka, Bangladesh

## Abstract

A large array of objects is networked together under the sophisticated concept known as the Internet of Things (IoT). These connected devices collect crucial information that could have a big impact on society, business, and the entire planet. In hostile settings like the internet, the IoT is particularly susceptible to multiple threats. Standard high-end security solutions are insufficient for safeguarding an IoT system due to the low processing power and storage capacity of IoT devices. This emphasizes the demand for scalable, distributed, and long-lasting smart security solutions. Deep learning excels at handling heterogeneous data of varying sizes. In this study, the transport layer of IoT networks is secured using a multilayered security approach based on deep learning. The created architecture uses the intrusion detection datasets from CIC-IDS-2018, BoT-IoT, and ToN-IoT to evaluate the suggested multi-layered approach. Finally, the new design outperformed the existing methods and obtained an accuracy of 98% based on the examined criteria.

## 1. Introduction

Rapid product introductions and great hopes the developing Internet of Things (IoT) technologies are currently being seen around the world. It is expanding quickly and connecting billions of gadgets that we use every day. According to a Gartner event analysis, by 2020, there will be almost 25 billion connected things [1]. These interconnected devices improve daily tasks and create clever solutions. However, the enormous benefits and prospects provided by IoT technology are overshadowed by serious security issues and privacy trade-offs [2]. There are a lot of factors to consider when designing solutions for the Internet of Things, including the sheer number of interconnected gadgets, the level of complexity involved, the prevalence of competing trends, and the wide range of variables that must be managed. The present security methods are only suitable for brief sessions on powerful computers [3].

The same method of protection cannot be used for prolonged sessions. These factors made IoT devices appealing targets for hackers, putting our lives in jeopardy from unforeseen dangers [4]. Using the terms “lightweight” and “adoption” to create strong security solutions could be one realistic way to handle these IoT complexity issues [5]. Solutions that are “adaptive lightweight” have repeatedly shown their value in resolving inconsistencies in very large distributed networks. The vast number of connected devices makes it extremely difficult to individually secure each one. There is a greater practical need to protect information passed between devices in an Internet of Things network [6–8].

Artificial intelligence can analyze a wide range of types and sizes of data and offer adaptive IoT system solutions. Massive amounts of IoT data are currently being analyzed using machine learning and data analytics techniques to enhance customer service and network performance. This study proposes a multilayered security method for an IoT system. Deep learning techniques were then used to monitor the IoT network in order to categorize activities as “normal” or “malware” for each tier of the design by establishing a baseline with the intrusion detection datasets from the CIC-IDS-2018, BoT-IoT, and ToN-IoT.

Deep learning has huge potential to draw insights from IoT data, even if it is still being studied in the IoT business, particularly in IoT security. We think that IoT solutions can be maximized through the clever application of deep learning techniques. Despite the complexity of the neural network topologies, lightweight functionality for IoT solutions can be obtained by tuning the hyperparameters. We based our approach on applying deep learning ideas to IoT network security on this supposition.

### 1.1. Aim of the Study

- To examine security concerns in the IoT context.
- To create a multilayered security solution for the transport layer in the Internet of Things network.

We anticipate that these findings will pave the way for future deep learning applications in fields like cybersecurity and the Internet of Things. The vast amount of heterogeneous data that must be explored to fully understand the importance of security in today's connected world necessitates the use of artificial intelligence. While many deep learning algorithms exist, the study problem calls for one with the ability to glean insights from past data. In light of this, we choose to employ neural networks in our research. With this detailed introduction, Section 2 examines the background work, Section 3 describes the system methodology, Section 4 explains the experimental design, followed by a conclusion in Section 5.

### 1.2. Related Study

The Internet of Things (IoT) concepts and technology, as well as its security and privacy problems, are covered in this section. This section also discusses the qualities that should be taken into account when creating IoT security solutions. In this part, network security and intrusion detection technologies are further explained. We describe the network's structure and illustrate the significance of ML and DL in IoT security with real-world use cases.

The IoT is made possible by the fusion of numerous enabling technologies. Significant IoT contributors include sensors, smart technologies, nanotechnologies, and radio frequency identification (RFID). Wireless microchips, known as RFID devices, are used to automatically identify and tag items. With the help of tags to feel the collections and detect the channel, these devices can wirelessly identify an object even when it is out of line the of sight. Credit cards, automotive ignition keys, and other modern applications all make use of RFID technology. In order to use mobile nodes and build intelligent systems, RFID needs to be used in the Internet of Things [9].

Adaptive technology with reliable network performance, gadgets like smart appliances, smartphones, and other wearable technology enable the Internet of Things (IoT) dream. The IoT system's resources can be accessed through smart technologies, which also increase the network's processing power [10]. Nanotechnologies are used by complex IoT systems and have the ability to influence the development of intelligent solutions. Nanosensors, for instance, can be employed in urban settings to track the spread of illnesses. Although the Internet of Things (IoT) has numerous advantages for society, it also raises significant privacy and security issues. Because it relies on real-time apps and the fact that the vast majority of IoT devices are left unattended without any form of monitoring, the IoT system presents several privacy and security challenges. There are a wide variety of Internet of Things-related infrastructure, network, device, and interface vulnerabilities [11].

The sheer variety and number of nodes in an IoT network make it difficult to implement per-device security. Data transmissions on a network can be monitored to detect intrusion attempts. If you are concerned about the security of your IoT devices, you might want to consider network-based solutions instead, as they only require minor tweaks to work with different networks. Devices on an IoT network need to be registered before they can gain access or be protected from intrusion. Each device's whole incoming and outgoing traffic must be watched, and a template for the typical network traffic flow must be developed. Any network data that deviate from the expected behavior is considered a threat, and the device owners are alerted [12].

An intrusion detection system (IDS), a piece of software that keeps an eye on harmful activity on networks or systems, can assist in achieving network security. IDS can be divided into various types. IDS are divided into active IDS and passive IDS categories based on how responsive they are [13]. The IDS is also divided according to where it is mounted. IDSs are referred to as “network intrusion detection systems” when installed on a network segment and “host-based intrusion detection systems” when installed on workstations. There are many problems with host-based intrusion detection systems, and they might not be appropriate for research [14].

The main difference between deep learning and machine learning is how performance varies as data amount increases. Machine learning uses the least amount of data while deep learning algorithms use more data to detect patterns in the network. In the study of different, multimodal IoT data, deep learning is also applicable [15]. Traditional machine learning algorithms cannot deliver long-term results for IoT devices, which are frequently connected for prolonged periods. The performance of the method can be significantly impacted by various deep neural network topologies [16]. A multilayer neural network is a deep structure that may be created by stacking the network with numerous layers. This method has several uses in high-dimensional data, weather forecasting systems, and speech recognition systems [17]. To achieve optimal performance, multi-layered neural networks share hyperparameters, weights, and biases throughout all of their layers.

## 2. System Methodology

The key characteristics required for IoT solutions include learning from the past, being lightweight, multilayer, distributive, and adaptive. In order to scale down the size of the datasets used by the IDS classifier, we developed a unique IoT network architecture. To conduct our experiments, we chose the CIC-IDS-2018, BoT-IoT, and ToN-IoT Intrusion Detection Datasets [18]. Using a decision tree classifier, we did feature engineering and chose the features that were of the utmost value. Before the data were utilized as input to the model, we thoroughly analyzed the data and produced it necessarily. Multimodal data are sent over time in an IoT system, which is built with a variety of heterogeneous devices. We identified the following three crucial aspects as being precisely necessary to manage IoT systems:

### 2.1. Security

A few IoT devices have low-end operating systems, which prevents them from processing antimalware software. They cannot carry out complex malware protection measures, and they also do not have enough memory to hold the malware databases that are always growing. By utilizing security solutions, developers may more simply roll out security updates and gather data on device performance to assess whether new services or products are required to improve performance.

### 2.2. Multiple Layers

The varied capabilities of IoT end devices underscore the idea of a multi-layered distribution approach in the IoT architecture. The system is strong because of the multilayered architecture that handles devices and their data at many levels. A multilayered design that is dispersed across the system enables processes to operate at various levels, from complex to basic, depending on the situation. A single-layer approach may restrict the position or range of components and may not offer the best performance in an IoT system.

### 2.3. Maintenance

Compared to traditional handheld consumer devices, the Internet of Things has various maintenance needs. In an IoT context, it is expensive to keep track of the deployment's maintenance for a long time. Additionally, security solutions should be able to support evolving malware threats over time when used for longer periods.

## 3. Proposed Design

In light of the aforementioned needs for an IoT security solution, we have designed an architecture to provide security on intrusion detection activity. The topology of the neural network, which describes the number of layers and neurons for each layer with connections, is developed to show how feature extraction may be applied to an IoT network. The artificial neurons use forward propagation, which has a perceptron classifier and an activation function. IDS will gather all data traveling through a network node once it is installed, classifying it as either “attack” or “normal” and recording its classification. Due to the inherent diversity of smart IoT network systems, this strategy might not be effective. For this reason, we designed a multitiered neural network architecture that performs better over a longer period of time.

**Figure 1:**
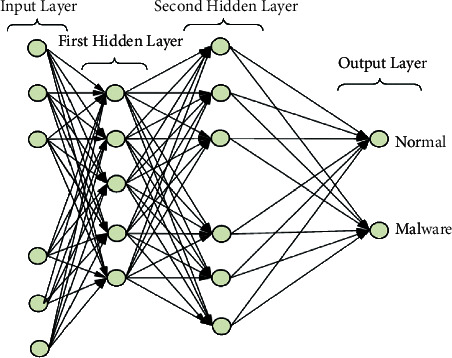
Neural network layers.

**Figure 2:**
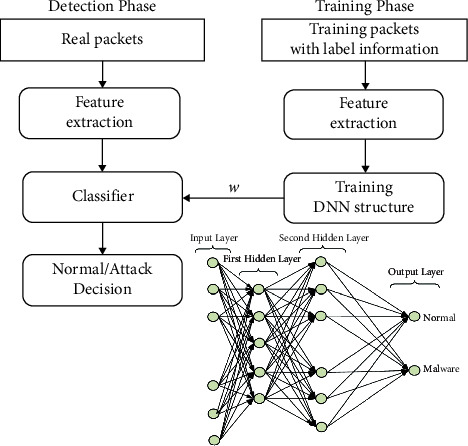
Multilayer architecture for IoT networks.

A single IDS system needs to be able to process network data from all linked devices fast and with appropriate memory. Since there are so many devices and they are so far apart, an IoT network will not function well under these conditions. Based on malware attacks that take place at the transport layer, we developed an architecture that enables four intrusion detection systems to take the place of a system-wide IDS. Each IDS positioned at a transport layer only keeps track of the information collected from the devices that are a part of that layer. By sharing the network burden with the system, the response time will increase.  Figure 1 depicts the neural layers and Figure 2 depicts the multilayered security architecture.

### 3.1. Feature Extraction

It is critical to limit the number of features and employ only those crucial features needed for the algorithm's training and testing. As a feature selection methodology, we employed a decision tree classifier, which has been shown to be the most effective way to reduce the dimensionality of datasets. The decision tree employs tree-based techniques that prioritize the value of the attributes in accordance with their capacity to enhance node purity (Gini impurity). Before entering the top 10 features for each dataset into the model, we graphically showed the significance of each feature. The input data's 92 features were reduced to 10 so that the model could be trained and used more quickly, making it versatile and adaptive.

### 3.2. Algorithm

- Training sample set as input.
- Set the feature ordering set and the original feature set *f* = *f*_0_,*f*_1_,*f*_2_,…to their initial values.
- The classifier in the decision tree has been trained.
- By using the F-test (ANOVA), it is possible to identify the features of a single variable.
- Determine the ranking score.
- The least-cored feature should be located.
- Refresh feature set *f*.
- Remove additional components from *f*.
- end for
- Output: Set *f* for feature sort.

### 3.3. IDS—Datasets

The CIC-IDS-2018, BoT-IoT, and ToN-IoT databases are the three most frequently utilized datasets. The testing data were gathered for three weeks, whereas the training data were gathered for ten. The total dataset includes more than 750 instances of IoT packet traffic and 84 different forms of network-based attacks. Either “normal” or one of the attack types is assigned to all network traffic. Links to the three different versions of the dataset are available in the repository on the Kaggle website, where the datasets are also available. Out of these three, the 20 percent CIC-IDS-2018 dataset is utilized most frequently in literature; hence we are using it in our study.

As was previously said, using the same dataset as before will allow us to compare the findings of this study to those of previous studies. There are 56 attack kinds in 20 percent of the CIC-IDS-2018 dataset, which is frequently referred to as malware. In all three datasets used for this study, seven attacks on transport layers are taken into account. A label of either “normal” or “malware” and 92 attributes are used to represent the training and testing samples. The functions can be broken down into three categories: those that reveal details about the command used to establish a connection; those that discuss the specifications of that command; and those that reveal details about other connections that point to the same destination and use the same service. We looked at every single piece of data available for this study.

## 4. Experimental Analysis

As can be seen in the architectural diagram, the dataset is segmented into various tiers depending on the nature of the assaults against the various TCP/IP layers. Since no attacks in the dataset can be classified as Link Layer attacks, this layer is ignored. Each kind of assault in the dataset belongs to the group depicted in Table 1 below, which describes the transport layer.

Based on the type of assault, each sample is read and added to a new data collection. There are a total of 472,454 samples in the CIC-IDS-2018 dataset for transport layer IDS, out of which 86,352 are considered “normal” and the other samples fall into one of seven attack categories. There are a total of 328,892 samples in the BoT-IoT dataset for the transport layer, of which 98,642 are classified as assaults. There are a total of 426,534 typical cases and 90,326 attack samples in the ToN-IoT dataset for the transport layer and all layers. In order to feed into the algorithm model, the three categorical elements from the dataset must first be transformed into numerical form. “Protocol type”, “service” and “flag” are functions that are encoded in numbers.

Every dataset has two portions: the training portion, which comprises 75% of the data, and the testing portion, which comprises 25% of the data. Later, a feature set and the related label set are created for each dataset. The labels “normal” and “malware” are encoded as [0 1] and [1 0], respectively. Using a multilayered neural network, the transport layer IDS classifier's full results and evaluation metrics are discussed. We began the tests by building a neural network with two hidden layers. For each hyperparameter set (learning rate, time-steps, and hidden layers), we carried out 25 sets of experiments and fine-tuned them to produce the desired outcomes. Because of this, we evaluated categorization using metrics including accuracy, precision, recall, and F-Score.

### 4.1. Feature Description

The features that were picked for the transport layer classifier are shown in Table 2. As can be seen from the table, all intrusion detection layers have the “protocol type” feature chosen. This indicates that the “protocol type” element provides sufficient information to categorize the label as “normal” or “malware.”

**Figure 3:**
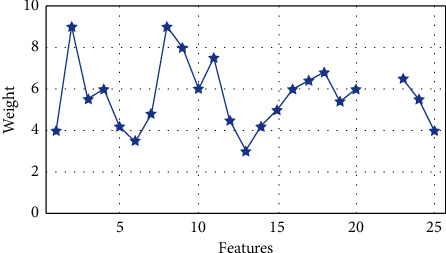
Weight for each input feature.

**Figure 4:**
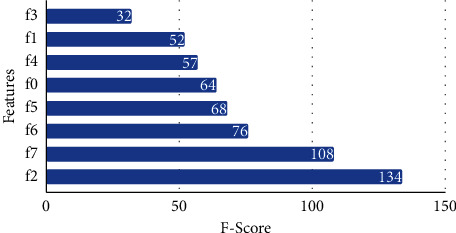
Feature importance for the transport layer.

The sample set of attributes and weight are shown in Figure 3. IDS has the following key attributes: 2, 8, 9, 11, 17, 18, and 23. This list serves as the input for our classification tasks.  Figure 4 shows the importance of the features for the transport layer, respectively.

### 4.2. Classifier Performance

Changing the hyperparameters of the neural network algorithm improves the performance of the Transport Layer IDS classifier. Training accuracy, recall, precision, and F-Score were compared to examine the model's responsiveness to iterative improvements. For the purpose of this experiment, we disguised two levels of encryption to meet the stringent security standards of the IoT platform. Results are summarized in Table 3.

**Figure 5:**
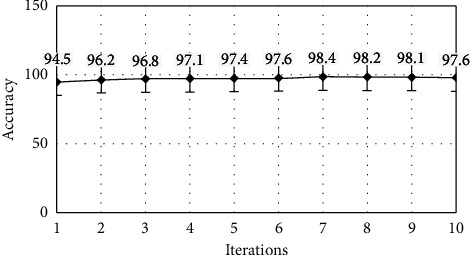
Accuracy analysis.

**Figure 6:**
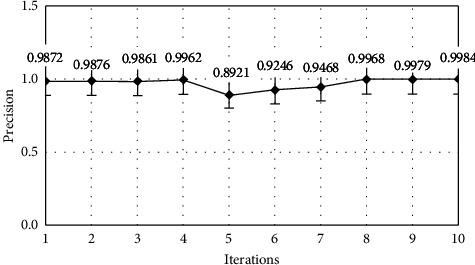
Precision analysis.

**Figure 7:**
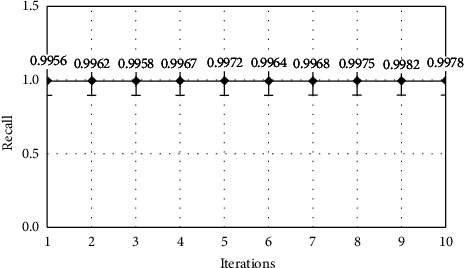
Recall analysis.

**Figure 8:**
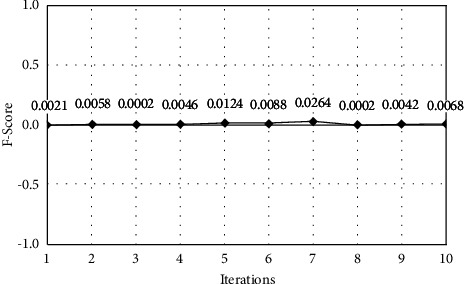
F-score analysis.

The model performance is optimized when the iteration count is set to 7, as seen in Table 3. Plots for the effects of iterations on the transport layer IDS classifier's accuracy (Figure 5), precision (Figure 6), recall (Figure 7), and F-Score (Figure 8) can be shown.

**Figure 9:**
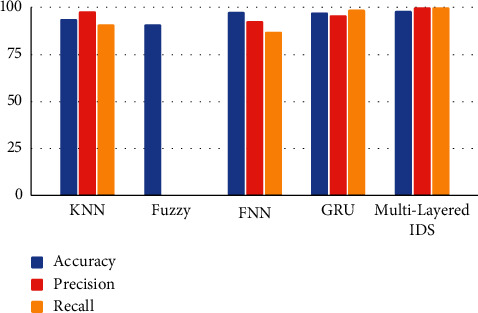
Existing methods Vs multilayered IDS.

The experiments in this section made use of the transport-layer attack dataset created in the previous section. The transport layer IDS classifier's optimized findings function better when used in a multi-layer design, making them appropriate for an IoT system.  Table 4 displays the results of our extra analysis, and we contrasted them with earlier research on the classification of intrusion detection using machine learning techniques.  Figure 9 demonstrates how our method outperformed all prior research attempts. We assume *X* patterns exist in the training set and Y in the validation or testing set. The multilayered network's (two hidden layer) neural net is fed pairs of patterns from the training set X. Each pattern is processed in parallel at the level of individual neurons using distributed (and asynchronous) processing. The initial weight and the features of the dataset also affect the convergence time, which is a random variable with a value that determines how many iterations are needed to get a solution. It is usual for the number of iterations to fluctuate slightly. The number of hidden layer neurons varies from dataset to dataset because of the varying number of patterns present.

## 5. Conclusion and Future Work

The significant essence of this work lies in the fact that deep learning techniques are being used to secure the IoT. Prior to tackling the security concerns of the IoT, we first took a close look at its underlying design. We focused our examination exclusively on data security in networks as part of our study. To detect intrusions in IoT networks, we have presented a multilayered neural network architecture. We proposed placing the IDS classifier at the transport layer based on the attack types seen there and the design of the layer itself. Because of this, the training set for the classifier shrunk, but accuracy, recall, precision, and F-score all improved. This strategy has produced excellent outcomes that outperform previous research in the literature. Additionally, we conducted the experiments using the CIC-IDS-2018, BoT-IoT, and ToN-IoT datasets. According to experimental data, the Transport Layer IDS outperforms all other IDS classifiers with an accuracy of 98.1 percent. In order to protect against security threats, it is critical to create robust solutions as the IoT deals with user personal data and industry information. Given that the Internet of Things produces a vast amount of heterogeneous data, this is conceivable using deep learning ideas. Convolutional and recurrent neural networks can be combined to create a hybrid network that can handle multimodal data. The IoT devices with limited processing power and small data sizes were the focus of this study. Applying this research to a sizable amount of real-time IoT data will advance it.

## Figures and Tables

**Table 1:** Attack Types for the transport Layer.

No	Type
1	Session hijacking
2	SYN flooding
3	TCP/UDP flood
4	Desynchronization
5	IPSec flood
6	False message Injection
7	Energy drain

**Table 2:** Transport layer features.

No	Selected features
1	Frame length
2	Header length
3	Number of packets
4	Protocol type
5	Error rate
6	Port rate
7	Service rate
8	TCP flags
9	src_host
10	dest_host

**Table 3:** Performance metrics for the transport layer IDS classifier.

Iteration	Accuracy	Precision	Recall	F-score
1	94.5	0.9872	0.9956	0.0021
2	96.2	0.9876	0.9962	0.0058
3	96.8	0.9861	0.9958	0.0002
4	97.1	0.9962	0.9967	0.0046
5	97.4	0.8921	0.9972	0.0124
6	97.6	0.9246	0.9964	0.0088
7	98.4	0.9468	0.9968	0.0264
8	98.2	0.9968	0.9975	0.0002
9	98.1	0.9979	0.9982	0.0042
10	97.6	0.9984	0.9978	0.0068

**Table 4:** Existing IDS classifiers Vs proposed IDS classifier.

Method	Accuracy	Precision	Recall
KNN	93.6	98	91
Fuzzy	91	—	—
FNN	97.4	92.5	86.9
GRU	97.1	95.8	98.7
**Multilayered IDS**	**98.1**	**99.8**	**99.8**

The bold values indicate highest accuracy, precision, and recall values.

## Data Availability

The data used to support the findings of this study are included in the article.
